# Supplementary material for: Recurrence in the evolution of air transport networks
Source: Sci Rep. 2021 Mar 9;11:5514. doi: 10.1038/s41598-021-84337-z (PMC7943603; doi:10.1038/s41598-021-84337-z)
Supplement: Supplementary file 1 — Supplementary Information. [file 41598_2021_84337_MOESM1_ESM.pdf]

Supplementary Information: Recurrence in the evolution of air transport networks

Kashin Sugishita and Naoki Masuda

Results for different network distance measures

We analyze distance matrices for each carrier using three network distance measures in this section. The adjacency spectral distance between two networks G and G' is defined as

$$d_A(G,G') = \sqrt{\sum_{i=1}^{\max\{N,N'\}} (\lambda_i^A - \lambda_i^{A'})^2}, \tag{S1}$$

where N and N' are the number of nodes in G and the number of nodes in G', respectively; λ_i^A and $\lambda_i^{A'}$ are the ith largest eigenvalue of the adjacency matrix A for G and the adjacency matrix A' for G', respectively[1]. We pad $\lambda_i^A (i = N+1, \ldots, N')$ with zero values if $N < N'$ and similarly if $N > N'$. The other two network distance measures use different types of Laplacian matrices[2–4]. The combinatorial Laplacian matrix is defined by $L = D - A$, where D is the diagonal matrix whose ith diagonal element is equal to the degree of node i. The normalized Laplacian matrix is defined by $L' = D^{-1/2}LD^{-1/2} = I - D^{-1/2}AD^{-1/2}$, where I is the identity matrix. Using the eigenvalues of the combinatorial Laplacian matrix and those of the normalized Laplacian matrix, we define the combinatorial Laplacian spectral distance (which we simply refer to as the Laplacian spectral distance) and the normalized Laplacian spectral distance, respectively, in the same manner as Eq. (S1)[5,6].

We show the distance matrices obtained from the three network distance measures in Supplementary Fig. S1. With these three network distance measures, the distance matrix does not present sudden changes in response to the post-bankruptcy mergers for the three FSCs (i.e., the merger between American Airlines and US Airways in July 2015, that between United Airlines and Continental Airlines in January 2012, and that between Delta Air Lines and Northwest Airlines in January 2010). By contrast, the distance measure used in the main text shows clear horizontal or vertical lines that correspond to the sudden changes triggered by the three mergers (Fig. 2).

References

1. Wilson, R. C. & Zhu, P. A study of graph spectra for comparing graphs and trees. *Pattern Recognit.* **41**, 2833–2841 (2008).
2. Chung, F. R. & Graham, F. C. *Spectral Graph Theory* (American Mathematical Society, Providence, RI, 1997).
3. Cvetkovic, D. M., Rowlinson, P. & Simic, S. *An Introduction to the Theory of Graph Spectra* (Cambridge University Press, Cambridge, 2010).
4. Masuda, N., Porter, M. A. & Lambiotte, R. Random walks and diffusion on networks. *Phys. Rep.* **716–717**, 1–58 (2017).
5. Wills, P. & Meyer, F. G. Metrics for graph comparison: A practitioner's guide. *PLOS ONE* **15**, e0228728 (2020).
6. Masuda, N. & Holme, P. Detecting sequences of system states in temporal networks. *Sci. Rep.* **9**, 795 (2019).

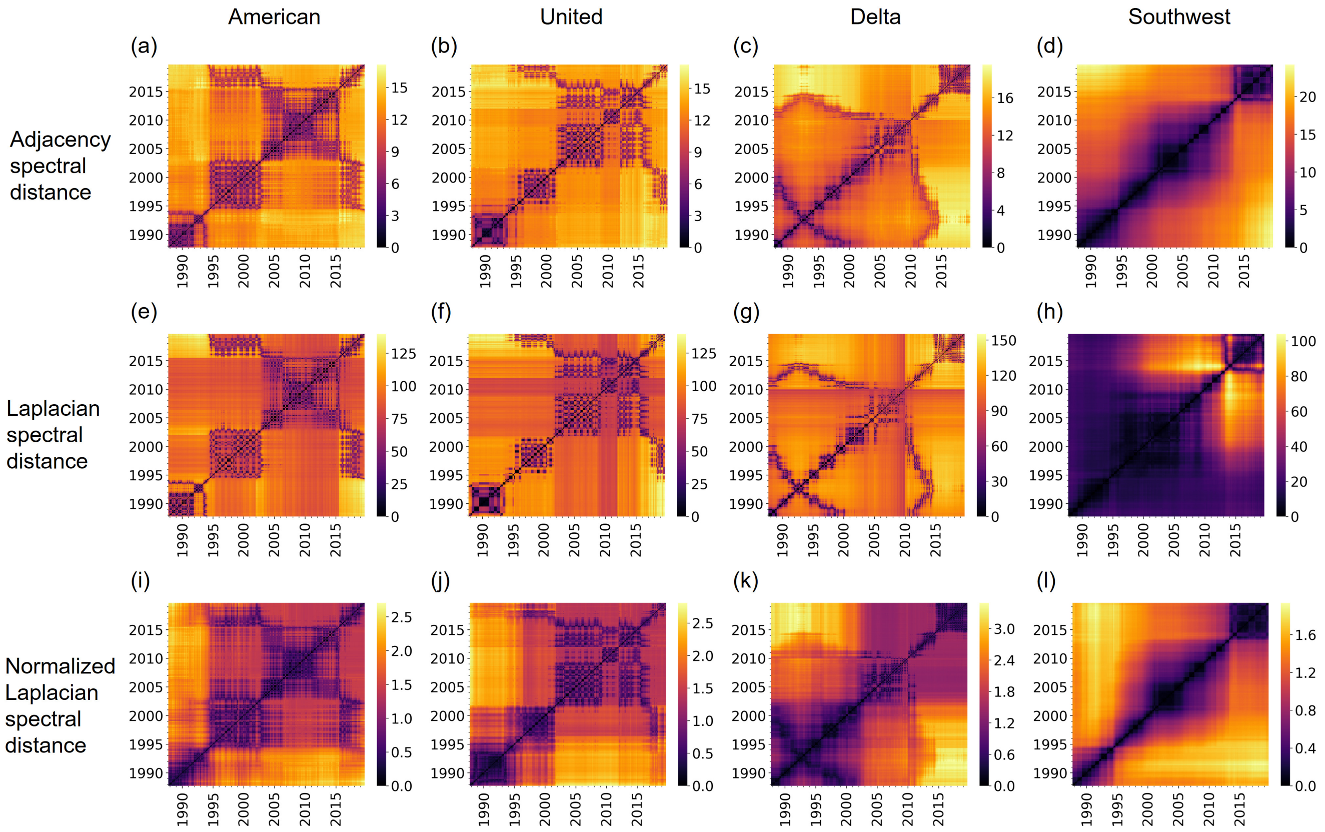

Supplementary Fig. S1. The distance matrices for the four carriers based on three network distance measures.
